# Reference Results for Blood Parameter Changes and Recovery after Pelvic Radiation without Chemotherapy

**DOI:** 10.3390/hematolrep14020023

**Published:** 2022-05-12

**Authors:** Gregory P. Swanson, Kendall Hammonds, Sameer Jhavar

**Affiliations:** Baylor Scott and White Health, Temple, TX 76508, USA; kendall.hammonds@bswhealth.org (K.H.); sameer.jhavar@bswhealth.org (S.J.)

**Keywords:** blood counts, WBC counts, hematotoxicity

## Abstract

Introduction: There are few reports on the effect of radiation alone on blood cells (without chemotherapy). We sought to develop a single source as a reference. Materials and Methods: For over 300 prostate cancer patients treated with radiation alone, we collected the baseline, end-of-treatment and three-month post-therapy complete blood counts (CBC). Results: The hemoglobin dropped by a mean of 1.00 g/dL (−7.1%), with an RBC count of 0.40 × 10^12^ (−8.6%) at the end of treatment and remained significantly (but <5%) below baseline at follow-up. Significant declines were seen in the levels of the granulocytes (−12.2%; −0.67 × 10^9^), monocytes (−2.2%; −0.05 × 10^9^) and platelets (−12.7%; −30.31 × 10^9^) at the end of treatment, but all returned to baseline on follow-up. The neutrophils and basophils (the primary components of the granulocytes) suffered a significant decline but returned to baseline by the follow-up. The other granulocyte components, the eosinophils, did not decline significantly. The most dramatic decline was in the levels of lymphocytes −62.5% (−1.29 × 10^9^), which were still significantly below baseline (−38%) after two years. Conclusion: The effect of radiation is mostly transitory, with some persistence in hemoglobin/erythrocyte levels (<5%). Lymphocytes are slower to recover, remaining significantly below baseline after two years. It is noteworthy that of the patients whose lymphocytes were in the normal range at the start of therapy, only 14% were below normal at follow-up. Radiation alone has negligible-to-modest long-term effects on blood counts.

## 1. Introduction

Radiation has been reported to have an effect on blood parameters, including erythrocytes (red cells), white blood cells (granulocytes (neutrophils, eosinophils, basophils) and lymphocytes), and platelets. Most radiation therapy is given with chemotherapy; since chemotherapy has a direct effect on all blood components, the specific effect of radiation is not well defined. We report a study of the effect of pelvic radiation on blood counts in over 300 prostate cancer patients without the confounding effect of chemotherapy. This resulted in a specific examination of the effects of radiation on blood counts and their recovery.

## 2. Materials and Methods

The utilization of intensity-modulated radiation therapy (IMRT) results in a wider distribution of dose throughout the normal tissue than previous techniques. We started to routinely collect complete blood counts (CBC) in late 2014 to monitor the effect of the broader dose distribution. We prospectively obtained a complete blood count at baseline, the last week of treatment and three months later. Subsequently, having detected no obvious clinically relevant detrimental effect on blood counts, we terminated the routine collection. A subgroup patients sporadically provided blood counts later on, and these results were collected (mean follow up 28 months). After obtaining institutional review board (IRB) approval, we evaluated the collected data to examine the effect of the radiation treatment on the blood counts.

The mean age of the 301 patients was 69.3 years. Patients consisted of those receiving primary prostate radiation therapy (*n* = 193) or those receiving post-radical-prostatectomy radiation therapy (*n* = 108). All patients were treated with intensity-modulated radiation therapy (IMRT). Initial treatment was at 1.8 Gy/day with final prostate/prostate fossa boost dose at 2 Gy/day. The majority (83%) received lymphatic (pelvic) radiation at a dose of 54 Gy. The primary prostate patients usually received total dose of 78 Gy, although some (*n* = 22) received external beam therapy followed by an implant boost and a few rare patients in this time period underwent hypofractionated radiation therapy (*n* = 3). One patient underwent hypofractionated boost. Post-operative patients received 70 Gy to the prostate fossa. Fifty percent of the patients received concomitant androgen ablation, which on prior review did not have an acute effect on the blood components. [1] The pelvis was defined as including L4–5, sacrum, bilateral os coxae and upper femurs (down to the level of the inferior ischium). Previous experience has shown that the volume of the radiation field is a significant factor in blood count effect, so we divided our patients based on whether they received radiation to the pelvis (with a subsequent boost) or prostate/prostate fossa only. Not all patients had normal blood counts at baseline (according to our laboratory parameters), and to avoid confounding the results, we also evaluated those that were in the normal range separately.

Sample characteristics were described using descriptive statistics. Frequencies and percentages were used to describe categorical variables, including the number of patients who fell within normal ranges for each CBC component. Means and standard deviations were used to describe continuous variables, including the percentage change in each CBC. Changes in blood counts were measured in two ways: absolute change from baseline and percentage change from baseline. Absolute change was calculated at each time point by subtracting the baseline value from the value at that time point. Percent change was calculated by taking absolute change, dividing by the baseline value, then multiplying by 100. Wilcoxon signed-rank tests were used to determine whether the percentage change in blood counts at each timepoint was significantly different from no change (0%). Chi-square tests were used to test for differences in normal blood count rates between patients who received lymphatic radiation and patients who did not receive lymphatic radiation. Statistical significance was determined by *p*-values less than 0.05. All statistical analyses were performed in SAS 9.4.

## 3. Results

**Figure 1:**
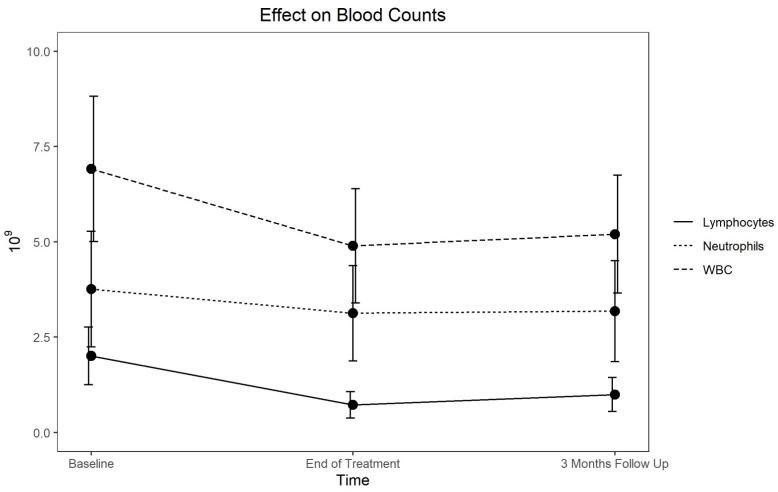
Response and recovery of lymphocytes, neutrophils and WBC.

For the entire cohort (Table 1), the hemoglobin level, red blood cell (RBC) count, white blood cell (WBC) count, granulocytes, lymphocytes and platelets declined by the end of the treatment, with the eosinophils, basophils and monocytes relatively unchanged. The platelet counts declined but recovered fully. Specifically, the hemoglobin levels dropped by a mean of 1.00 g/dL (−7.1%) and the RBC count by 0.40 × 10^12^ (−8.6%) at the end of treatment and then slowly improved. At baseline, the mean hemoglobin level was at the lower limit of normal, at 51% below normal. For those in the normal range, at the end of the treatment, 59% dropped below normal and were slow to recover (Table 2). The changes in the levels of WBC, lymphocytes and neutrophils are shown in Figure 1.

The granulocytes declined by 12.2% (−0.67 × 10^9^) and the lymphocytes by 62.5% (−1.29 × 10^9^) (Table 1). At follow-up, the granulocytes completely recovered, although the lymphocyte recovery was more gradual. For the patients who had normal levels at baseline, 5% of the granulocyte counts and 14% of the lymphocyte counts remained below normal (Table 2).

Given the likely correlation between the treatment volume and the fall in the blood counts, we evaluated the effect of the field size. The mean/median volume of the pelvic bones in our patients was 1670 cm^3^. As expected, the incremental dose per volume was much less for the prostate/prostate fossa fields than for those preceded by whole-pelvis treatment. For example, more pelvic bones (55%) received 20 Gy with the large field than the small field (32%). These differences were consistent across all the dose levels (Table 3).

With the different field sizes, the smaller field sizes had less of a detrimental effect on the counts (Table 4). The hemoglobin dropped by a mean of 1.10 vs. 0.51 g/dL, the RBC by 0.44 vs. 0.19 × 10^12^, the granulocytes by 0.74 vs. 0.31 × 10^9^, the lymphocytes by 1.34 vs. 1.02 × 10^9^ and the platelets by 33.8 vs. 12.4 × 10^9^ for the large versus the small fields, respectively. Again, the eosinophils, basophils and monocytes did not display any significant changes with any of the fields. The platelets and granulocytes recovered completely, but the Hgb, RBC count, and lymphocyte counts were incomplete and lagged behind for the larger fields. For the patients who started in the normal range (Table 5), the hemoglobin/RBC, granulocytes and lymphocytes had a significantly greater decline with the larger fields, while the eosinophils, basophils and platelets displayed no difference. With the longer follow-up, the field-size effect differences resolved for all the counts, except for the lymphocyte.

## 4. Discussion

The components of the blood are ubiquitous throughout all the tissues of the body—specifically the soft tissues, the bones (primarily the bone marrow), the vasculature (from which most measurements are made) and, in the case of white blood cells (especially for lymphocytes), the lymphatics. Radiation influences each of those compartments according to the treatment volume.

There appear to be differences between the radiosensitivity of mature cells and precursor cells [2]. When compared to lymphocytes, most mature cells are thought to be relatively radioresistant. All blood components originate from pluripotent hematopoietic stem cells, which differentiate into various cell lines. Except for some lymphocyte subgroups, all the cells primarily develop and mature in the bone marrow, although there is a small number of hematopoietic stem cells in the peripheral blood [3]. While there may be some variation between the various primordial cells [2,4] they are all considered radiosensitive, with a single dose of 6 Gy considered uniformly ablative. This is consistent with the earliest single doses to ablate bone marrow pre-transplant. To reduce toxicity, the single dose has been supplanted with fractionated whole-body radiation combined with chemotherapy, with 15 Gy considered the most effective [5]. In our patients, the entirety of the pelvis bone encompassed approximately 50% of the active marrow [6]. The percentages of our small- and large-volume treatments that received or exceeded 20 Gy were 61% and 34% (median) (Table 3), respectively, representing approximately 30.5% and 17% of the total active marrow, respectively.

In terms of the effect of radiation in vivo, the radiosensitivity of cells is mitigated by the body’s compensatory response. The compensatory response (uniformly mounted from the bone marrow) is tempered by the radiation’s effect on the supporting structures.

### 4.1. Erythrocytes (Red Blood Cells)/Hemoglobin

It has long been established that mature erythrocytes are acutely radioresistant [7], even up to doses exceeding 200 Gy. Lacking concise in vivo data, we rely on several observations. With extracorporeal radiation, the blood is radiated continuously and circulates back into the patient. Both normal senescence and compensatory mechanisms have an effect on the red cell count, and it has been found that after 20 days with extracorporeal blood doses of 350 Gy, the decline in labeled erythrocytes was 14% greater than the normal decline [8]. Most of the other data are from blood radiated ex vivo for storage. In a well-known study [9], the doses used were 50, 100 and 200 Gy for both whole blood and packed cells stored for 21 days. There was no difference in post-transfusion red cell survival between irradiated and unirradiated blood. It was noted that the half-life of the red cells radiated to 200 Gy was lower than normal, but not statistically significant. In a later study using 40 Gy before storage [10], samples of blood were tested every 7 days. The rate of lysis was very low (<1%), indicating little direct effect of the radiation. A later study [11], appeared to show that red cells are indeed damaged directly as potassium and free hemoglobin are increased and ATP is decreased (after storage). In spite of this, with 15 Gy delivered before storage, the red cell recovery for non-radiated cells was 84.5% and for radiated cells it was 81.2% (NS). In a contemporaneous study [12], cells were radiated with 30 Gy and their viability was measured after 42 days of storage. After transfusion, the authors found a decline in red cell recovery from 78.4% to 68.5% (*p* < 0.02), indicating a modest but significant drop in viability. Overall, the metabolites do suggest that there is some radiation effect, but the results on viability appear negligible. Therefore, any significant effect of radiation on erythrocyte counts in vivo would mostly be on precursor cells. We saw a modest ~1 gm/dL drop by the end of treatment. This is consistent with other radiation-only studies, in which hemoglobin declines by the end of treatment of 0.3–0.9 gm/dL were observed [13,14]. We saw at least partial recovery by 3 and 31 months (improving to −0.71 and −0.59 gm/dL below baseline, respectively) (Table 1). Even with a modest drop, a large number of patients fell out of the normal range (Table 2), which is likely to have reflected the lower counts at baseline in our elderly cohort, of whom 51% were already below normal. The field size had an effect on the recovery, with a persistent hemoglobin decline below the mean of −0.65 gm/dL for the large field and −0.25 gm/dL for the small field (Table 4). This is interesting in that there was <15% more active marrow treated with the larger fields.

### 4.2. White Blood Cells

Several studies report the effects of radiation on white blood cells. Considering that this includes a wide range of cells, including the highly radiosensitive lymphocytes, the information obtained is not as specific as the component analysis. Overall, we did see a decline in WBC counts (Table 1, Table 2, Table 4 and Table 5) and, in this article, we review the effect on the various components.

### 4.3. Granulocytes, Neutrophils, Eosinophils and Basophils

Granulocytes include of neutrophils, eosinophils and basophils. The acute direct effects of radiation on granulocyte function are also mitigated by immediate compensatory mechanisms in vivo. For example, a pool of marginal cells is rapidly mobilized with acute loss, which makes the direct effects of radiation on mature granulocytes in vivo difficult to measure. In vitro, in the evaluation of blood treated with 50 or 100 Gy and stored for 21 days, although not enumerated, the granulocyte function appeared to be unchanged [9]. It is not surprising that 20 Gy is not thought to damage granulocytes [15]. Overall, we saw the granulocyte counts drop by 12% (*p* < 0.0001) at the end of treatment, but they recovered at the subsequent follow-up (Table 1). In our experience, the eosinophils were not significantly changed (which is consistent with other reports [16]) and, initially, the basophils dropped significantly, recovered partially at 3 months and then recovered completely with the longer follow-up (Table 1). The neutrophils declined 11.2% by the end of the treatment (−0.63 × 10^9^/L) and remained 9.7% below baseline at 3 months (*p* < 0.0001), but also recovered with the longer follow-up. The post-treatment decline was similar to that reported by others [14,16,17], ranging from −0.7 to −2.2 × 10^9^/L.

### 4.4. Lymphocytes

Unlike other mature blood cells, mature lymphocytes are considered among the most radiosensitive of the mammalian cells. This has been well documented through extensive studies. The clinical data on the immediate effects of radiation on lymphocytes come from the bone marrow transplant experience, where the goal is to eradicate lymphocytes and their progenitors. The data on radiation effects is not “pure”, in that most patients are preconditioned by chemotherapy, which has significant effects on lymphocytes. With this caveat, the dose of 15 Gy over 3 days is felt to be effective, although lower doses (e.g., 12 Gy) are often used for lower toxicity [5]. It is not unreasonable to consider that without the addition of chemotherapy, that a fractionated dose of 20 Gy or more is necessary. The effect on lymphocytes is almost immediate. With just a single dose of 1.35–1.4 Gy, the lymphocyte count drops to 65% of pretreatment levels, mostly within the first 4 h [18]. While the volume of blood, lymphatics and bone marrow radiated remain critical, the acute radiosensitivity of mature lymphocytes makes the effect on circulating lymphocytes more significant than on other cells. This is demonstrated in the treatment of cancers in the chest. There is only a modest amount of active bone marrow, but the level of lymphopenia still correlates with the field size (i.e., vascular volume) [19]. Even with small volumes and minimal fractions, there is still a decline in lymphocytes [20]. Furthermore, given the sensitivity of circulating lymphocytes, the number of daily fractions has a greater cumulative effect than in other cell lines. Our patients experienced a 62.5% decline by the end of their treatment, and although they improved, the values were still below baseline (−38%, *p* < 0.0001) at 28 months. For the patients that were in the normal range at baseline, only 14% were below baseline at their last follow-up. Other studies are remarkably similar. In these studies, the baseline lymphocyte count is approximately 2.0 × 10^9^ (range 1.4–2.1 × 10^9^) and declines to approximately 0.7 × 10^9^ (0.46–1.0 × 10), representing a 60% decline [14,16,17,21].

### 4.5. Monocytes

There is little direct clinical information on the effect of radiation on monocytes. In vitro, monocytes treated with 25 or 50 Gy had decreased (30%) survival compared to non-irradiated controls. There was no difference between the 25 and 50 Gy cohorts [22]. Although it was small (−0.05 × 10^9^/L), we saw a significant drop in number by the end of treatment, which, in fact, worsened (−0.08 × 10^9^/L) at the initial 3-month follow-up, but then returned to normal after a longer follow-up (Table 1). The only other report showed a −0.7 × 10^9^/L decline at the end of the treatment [16].

### 4.6. Platelets

There is no acute destruction (lifespan-shortening) of platelets with doses up to 750 Gy [23]. Furthermore, most studies did not show a detrimental effect on their function [23]; however, one study found that when transfused, the yield of circulating platelets that had been radiated to 50 Gy was lower with a resulting functional deficit [9], although this was not seen with platelets radiated to 15 Gy [24], to 20 Gy [25], 30 Gy [12], or even 50 Gy in other studies [26]. In our patients, the platelet counts did decline modestly (−12.7%) and, although they were still significantly lower at 3 months (−6.2%, *p* < 0.0001), they returned to baseline at the subsequent follow-up (*p* = 0.1336). Every other study bar one showed a decline by the end of treatment, ranging from 22–87 × 10^9^ (9–33%) [13,14,17,21,27].

## 5. Conclusions

While radiation treatment has a negative effect on blood components, it is limited and mostly transitory. Every parameter other than eosinophils had a significant decline by the end of treatment (Table 1) and, while the parameters were all improved by 3 months, they were still significantly below baseline (<10%, other than the lymphocytes). After a further follow-up (mean 28 months), only the Hgb/RBC (at <−5%) and lymphocytes (−38%) were significantly below baseline. For the patients who started in the normal range, 41% still had a below-normal hemoglobin level, but only 14% had a lymphocyte count below normal. For every parameter, the patients treated with larger fields had greater declines, but after follow-up, other than the lymphocytes (with a difference in decline between the two field sizes of <12%), the differences in the other parameters were not significant.

## Figures and Tables

**Table 1:** Entire cohort (*n* = 301) effect on blood counts from baseline to end of treatment, 3-month follow-up and longer-term (“final”) follow-up (mean 28 months).

	Baseline Mean (SD)	EOT Mean (SD)	Percent Change from Baseline (SD)	3-Month Mean (SD)	Percentage Change from Baseline (SD)	*p* Value	Final Mean (SD)	Change from Baseline (SD)	*p* Value
Hgb	13.95 (1.32)	12.96 (1.33)	−7.1% (6.32)	13.24 (1.34)	−4.9% (7.17)	<0.0001	13.36 (1.51)	−4.0% (9.55)	<0.0001
RBC	4.65 (0.46)	4.26 (0.50)	−8.6% (6.56)	4.35 (0.47)	−6.6% (7.07)	<0.0001	4.44 (0.51)	−4.6% (8.94)	<0.0001
WBC	6.91 (1.91)	4.89 (1.50)	−27.6% (18.82)	5.20 (1.55)	−22.7% (18.32)	<0.0001	5.86 (1.70)	−12.4% (24.33)	<0.0001
Granulocytes	4.03 (1.52)	3.37 (1.24)	−12.2% (30.93)	3.43 (1.32)	−10.2% (29.74)	<0.0001	3.83 (1.47)	+1.4% (42.60)	0.1236
Eosinophils	0.23 (0.19)	0.21 (0.18)	+12.7% (98.66)	0.21 (0.14)	+15.2% (118.22)	0.2289	0.21 (0.20)	+15.7% (143.54)	0.1169
Basophils	0.04 (0.03)	0.03 (0.03)	−21.4% (61.74)	0.03 (0.03)	−7.4% (79.12)	0.0004	0.04 (0.03)	+28.4% (144.33)	0.0263
Neutrophils	3.76 (1.51)	3.13 (1.25)	−11.2% (41.24)	3.18 (1.32)	−9.7% (33.89)	<0.0001	3.58 (1.47)	+3.85% (53.74)	0.2711
Lymphocytes	2.01 (0.76)	0.72 (0.35)	−62.5% (15.95)	1.00 (0.44)	−48.8% (17.05)	<0.0001	1.18 (0.48)	−38.2% (24.27)	<0.0001
Monocytes	0.62 (0.21)	0.57 (0.20)	−2.2% (33.97)	0.54 (0.18)	−9.6% (29.23)	<0.0001	0.59 (0.20)	+1.4% (37.16)	0.1453
Platelets	218.20 (51.30)	188.95 (48.18)	−12.7% (16.99)	202.50 (46.18)	−6.2% (14.91)	<0.0001	214.29 (57.09)	−0.1% (22.39)	0.1336

EOT = end of treatment. Units of measurement: hemoglobin (Hgb): g/dL; red blood cell count (RBC): ×10^12^; all others: ×10^9^/L. SD = standard deviation.

**Table 2:** Normal blood count levels in our laboratory and changes with treatment.

Parameter	Normal Range	% in Normal Range	% in Normal * Range End of Treatment	% Normal * Range 3 Months	% Normal * Range Final
Hemoglobin	14–18 g/dL	49%	41%	57%	59%
Red blood cell count	4.7–6.1 × 10^12^/L	45%	38%	41%	52%
WBC	4–8–10.8 × 10^9^/L	85%	53%	67%	79%
Granulocyte	1.92–8.64 × 10^9^/L	95%	92%	93%	95%
Lymphocyte	0.72–4.32 × 10^9^/L	98%	40%	72%	86%
Monocyte	0–1.08 × 10^9^/L	96%	99%	99%	98%
Eosinophils	0–0.76 × 10^9^/L	98%	99%	99%	99%
Basophils	0–0.22 × 10^9^/L	100%	100%	100%	100%
Platelets	150–450 × 10^9^/L	94%	85%	93%	92%

Note: Granulocytes include neutrophils, eosinophils and basophils. * Those that were in the normal range at baseline.

**Table 3:** Percentage pelvic bone volume for every 10 Gy increment, i.e., V10 is the percentage of the pelvic bone volume receiving 10 Gy for those who received pelvis treatment (followed by prostate/prostate fossa boost) versus those who received prostate/prostate-fossa-only treatment.

	Pelvis/Lymphatic (Pelvic Bone)			Small Prostate/Fossa		
Gy	cc (SD)	Mean (%) (SD)	Median (%) (IQR)	cc	Mean (%) (SD)	Median (%) (IQR)
V10	1170 (333)	70 (18)	77 (69, 81)	887 (274)	53 (15)	58 (52, 62)
V20	919 (320)	55 (18)	61 (56, 66)	531 (206)	32 (11)	34 (30, 39)
V30	625 (253)	37 (14)	42 (37, 46)	280 (123)	17 (7)	18 (14, 21)
V40	359 (167)	22 (10)	24 (19, 27)	164 (73)	10 (4)	11 (8, 12)
V50	178 (86)	11 (5)	12 (8, 14)	103 (46)	6 (3)	7 (5, 8)
V60	25 (19)	2 (1)	1 (1, 2)	25 (12)	2 (1)	1 (1, 2)
V70	6 (9)	0 (1)	0 (0, 1)	48 (8)	3 (1)	1 (0, 1)

SD = standard deviation. IQR = interquartile range.

**Table 4:** Blood count changes based on field size. Large field indicates pelvis treatment covering the lymphatic (followed by a small field boost). Small field is treatment for the prostate/prostate fossa only.

Large Field	Baseline Mean (SD)	EOT Mean (SD)	Change from Baseline (SD)	*p*-Value	3 Month Mean (SD)	Change from Baseline (SD)	*p*-Value	Final Mean (SD)	Change from Baseline (SD)	*p*-Value
Hgb	13.96 (1.29)	12.87 (1.31)	−7.8% (7.3)	<0.0001	13.18 (1.38)	−5.3% (7.2)	<0.0001	13.31 (1.58)	−4.5% (9.7)	<0.0001
RBC	4.65 (0.46)	4.22 (0.50)	−9.5% (6.4)	<0.0001	4.31 (0.48)	−7.4% (7.0)	<0.0001	4.42 (0.53)	−5.1% (9.0)	<0.0001
WBC	6.86 (1.92)	4.71 (1.44)	−29.7% (18.3)	<0.0001	5.08 (1.57)	−23.9% (18.5)	<0.0001	5.74 (1.69)	−13.3% (24.6)	<0.0001
Granulocytes	4.01 (1.55)	3.28 (1.25)	−14.1% (31.1)	<0.0001	3.39 (1.37)	−10.5% (30.9)	<0.0001	3.77 (1.48)	+1.2% (44.2)	0.1092
Eosinophils	0.22 (0.16)	0.21 (0.18)	−12.8% (203.3)	0.3297	0.20 (0.13)	+17.8% (127.8)	0.1843	0.20 (0.21)	+18.3% (155.0)	0.1261
Basophils	0.04 (0.03)	0.02 (0.03)	−23.2% (61.4)	<0.0001	0.03 (0.03)	−7.0% (83.9)	0.0013	0.04 (0.03)	+28.6% (151.8)	0.0529
Neutrophils	3.75 (1.53)	3.05 (1.26)	−13.4% (42.5)	<0.0001	3.15 (1.38)	−10.0% (35.4)	<0.0001	3.52 (1.48)	+3.6% (55.9)	0.2111
Lymphocytes	1.99 (0.76)	0.64 (0.26)	−65.8% (13.3)	<0.0001	0.92 (0.39)	−51.7% (14.8)	<0.0001	1.12 (0.44)	−40.1% (24.1)	<0.0001
Monocytes	0.61 (0.21)	0.56 (0.20)	−2.7% (35.7)	0.0023	0.53 (0.18)	−9.3% (30.6)	<0.0001	0.59 (0.21)	+2.8% (38.9)	0.3535
Platelets	218.13 (51.25)	185.42 (46.51)	−14.2% (17.3)	<0.0001	201.47 (44.32)	−6.6% (15.9)	<0.0001	214.44 (57.30)	+0.1% (24.0)	0.1590
Small field										
Hgb	13.90 (1.44)	13.40 (1.35)	−3.8% (5.4)	<0.0001	13.56 (1.10)	−2.5% (6.6)	<0.0001	13.65 (1.13)	−1.4% (8.4)	0.0413
RBC	4.63 (0.47)	4.45 (.49)	−4.0% (5.3)	<0.0001	4.51 (0.41)	−3.0% (6.1)	<0.0001	4.53 (0.40)	-2.0% (8.4)	0.0092
WBC	7.16 (1.82)	5.79 (1.45)	−17.0% (18.1)	<0.0001	5.79 (1.31)	−16.7% (16.6)	<0.0001	6.44 (1.65)	−7.9% (22.4)	0.0163
Granulocytes	4.13 (1.42)	3.78 (1.13)	−2.6% (28.2)	0.3877	3.60 (1.00)	−8.9% (22.8)	0.0085	4.13 (1.41)	+2.3% (33.5)	0.9786
Eosinophils	0.28 (0.30)	0.24 (0.15)	+12.6% (71.1)	0.7849	0.23 (0.15)	+1.2% (38.6)	0.8001	0.22 (0.16)	+2.6% (55.9)	0.8364
Basophils	0.04 (0.02)	0.03 (0.02)	−11.9% (63.6)	0.1225	0.03 (0.02)	−9.3% (46.7)	0.1670	0.04 (0.02)	+27.7% (98.3)	0.2338
Neutrophils	3.81 (1.44)	3.52 (1.10)	−0.2% (32.0)	0.6851	3.34 (0.99)	−7.9% (24.9)	0.0329	3.86 (1.40)	+5.4% (41.2)	0.9059
Lymphocytes	2.14 (0.73)	1.13 (0.44)	−45.3% (17.6)	<0.0001	1.39 (0.51)	−33.3% (19.9)	<0.0001	1.47 (0.57)	−28.4% (22.8)	<0.0001
Monocytes	0.65 (0.20)	0.62 (0.17)	+0.3% (24.5)	0.8454	0.57 (0.18)	−10.9% (20.6)	0.0004	0.60 (0.19)	−5.6% (25.8)	0.1323
Platelets	218.56 (52.11)	206.46 (52.86)	−5.0% (13.0)	0.0003	207.44 (54.53)	−4.2% (8.32)	0.0024	213.57 (56.6)	-1.0% (11.9)	0.7657

Units of measurement: hemoglobin (Hgb): g/dL; red blood cell count (RBC): ×10^12^; all others: ×10^9^/L. SD = standard deviation.

**Table 5:** Field size effect on patients starting in the normal range.

Patients Starting in Normal Range			Small Field			Large Field			Small vs. Large Field *p*-Values			
Small Field	Large Field		Below Normal End of Treatment	Below Normal at 3 Months	Below Normal at Follow Up	Below Normal End of Treatment	Below Normal at 3 Months	Below Normal at Follow Up	At Baseline	Below Normal End of Treatment	Below Normal at 3 Months	Below Normal at Follow Up
52%	49%	Hgb	33%	25%	36%	64%	46%	42%	0.6755	0.0055	0.0551	0.5566
42%	45%	RBC	37%	32%	30%	67%	64%	51%	0.5918	0.0125	0.0078	0.0850
88%	85%	WBC	27%	18%	14%	51%	36%	22%	0.1020	0.0185	0.0294	0.3275
98%	94%	Granulocytes	0%	5%	4%	10%	7%	5%	0.6727	0.0476	0.5336	1.0000
98%	98%	Eosinophils	0%	0%	0%	1%	1%	17%	0.8135	0.4432	0.5378	0.6326
100%	100%	Basophils	0%	0%	0%	0%	0%	3%	na	0.6584	0.6642	0.6596
98%	98%	Lymphocytes	11%	2%	2%	69%	33%	1%	0.4521	<0.0001	<0.0001	0.0116
96%	96%	Monocytes	2%	0%	0%	0%	1%	3%	0.8520	0.1996	0.5360	0.2776
96%	94%	Platelets	7%	5%	11%	16%	8%	7%	0.6001	0.1033	0.4556	0.2864

Hgb = hemoglobin, RBC = red blood cells, WBC = white blood cells.

## Data Availability

Data available on request due to restrictions, such as privacy or ethical issues. The data presented in this study are available on request from the corresponding author. The data are not publicly available due to the lack of an accessible server.
